# Evaluation of gross energy concentration of neutral detergent fiber contained in feed and fecal samples

**DOI:** 10.3168/jdsc.2022-0345

**Published:** 2023-04-28

**Authors:** J.D. Stypinski, P.J. Kononoff, W.P. Weiss

**Affiliations:** 1 Department of Animal Science, University of Nebraska–Lincoln, NE 68503; 2 Department of Animal Sciences, Ohio Agricultural Research and Development Center, The Ohio State University, Wooster, OH 44691

## Abstract

Starch and NDF are usually assumed to contain the same concentration of gross energy (GE), but NDF is more variable in chemical composition and varies more in the extent of digestion. The variable chemical composition of NDF may have direct implications on dairy nutrition models that predict dietary GE and use this estimate for also predicting digestible energy. For example, when NDF is enriched in lignin and protein, the concentration of GE would increase, whereas NDF enriched in ash would have the opposite effect. Current nutritional models, such as the NASEM (2021) and CNCPS (6.55), assume a GE coefficient of 4.20 Mcal/kg for NDF. This study aimed to determine the heat of combustion of NDF and to consider if it is a contributing factor to the variance in digestible energy. To do so, NDF residues were isolated from 9 feed and 8 fecal samples and then combusted. Approximately 0.20 g of NDF residues from 16 feeds (corn silage, n = 2; grass hay, n = 2; alfalfa hay, n = 2; wheat straw, n = 1; cottonseed hulls, n = 1; soyhulls, n = 1; distillers dried grains with solubles, n = 1; and total mixed ration, n = 6) and 34 fecal samples were collected. A bomb calorimeter (Parr 6400 Calorimeter, Parr Instrument Company) was used to determine concentration of GE in each NDF residue sample. The GE concentration of feed NDF was observed to be 4.03 ± 0.245 Mcal/kg, which was similar to that of fecal NDF (3.94 ± 0.245 Mcal/kg). The lack of difference between feed and fecal NDF GE implies that digested NDF is of a similar GE concentration as total feed NDF and that current nutritional models are validated in their current approach in predicting digestible energy from NDF. However, our observed estimate of GE in NDF is lower than what is assumed and across feed types varied from 3.85 to 4.19 Mcal/kg.

## Highlights

• The GE concentration of NDF was observed to be lower than currently estimated.
• The variation in GE concentration among NDF residues was high.
• Feed, total mixed ration, and fecal NDF were of similar GE concentration.

## Graphical Abstract

**Figure:**
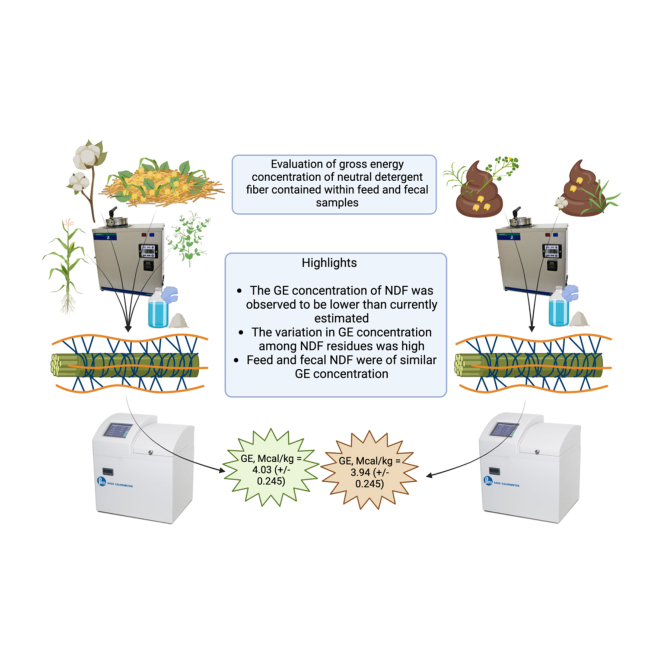
**Summary:** Dairy ration models predict the gross energy (GE) concentration of the diet using a 4.20 Mcal/kg coefficient for both feed neutral detergent fiber (NDF) and starch. The use of this GE concentration for starch has been validated; however, to our knowledge, the GE concentration of NDF has never been previously analytically determined using bomb calorimetry. The current study reports the GE concentration of feed NDF to be 4.03 ± 0.245 Mcal/kg. The high variation in GE concentration among feed NDF residues is likely a function of variable NDF chemical composition across individual feeds. The GE concentration of fecal NDF has also never been characterized. We hypothesized that the GE concentration of fecal NDF would be greater than the GE concentration of feed NDF because fecal NDF would be enriched in lignin; however, these were similar (averaging 3.94 ± 0.0245 Mcal/kg).

The gross energy (**GE**) concentration of a nutrient is a function of the elements present and the bonds that connect them (Hall et al., 2013). For homogeneous and uniform nutrients such as starch, variance around the true mean GE concentration is small. Kabo et al. (2013) reported the GE concentration of starch in feed is approximately 4.20 Mcal/kg with a coefficient of variation of less than 0.1%. Although the concentration of GE in NDF is assumed to be similar to starch, to our knowledge this has yet to be determined analytically. Underlining assumptions and analysis of the GE in dairy feeds is important because it is the starting point to estimate digestible energy (**DE**). Additionally, the mean concentration of GE in NDF could be subject to greater variation because, chemically, NDF is a heterogeneous fraction. For example, the carbon atoms in lignin are more reduced compared with other components of NDF; thus, lignin is associated with a greater GE concentration and believed to be 6.0 Mcal/kg (Voitkevich et al., 2012). Because lignin contains more GE than cellulose (4.15 Mcal/kg; Colbert et al., 1981) and xylan (the principal dimer of hemicellulose, 3.25 Mcal/kg; Gorensek et al., 2019), the GE concentration of NDF should increase when the concentration of lignin increases. Compared with feed, fecal samples from ruminants are generally enriched in lignin because lignin is not digestible (Hindrichsen et al., 2006). It therefore follows that fecal NDF residues should have a greater concentration of GE compared with feed NDF residues; however, this assumption fails to acknowledge that ash could also accumulate in fecal NDF residues and reduce its respective GE concentration. Thus, there is a need to analytically determine and compare the GE concentration of feed and fecal NDF residues. If the true concentration of GE in NDF is lower than that currently assumed, nutrition models may in turn overpredict the energy provided by NDF and this could potentially lead to formulations that are limited in energy. The objective of the current study is to analytically determine the GE concentration of feed NDF, and to compare it with that of fecal NDF. We hypothesize that fecal NDF will be enriched in lignin, and therefore contain more GE concentration, but that ash could also interfere with the estimate.

To evaluate the GE concentration of feed and fecal samples, approximately 0.20 g of NDF residues from 16 feeds [corn silage, n = 2; grass hay, n = 2; alfalfa hay, n = 2; wheat straw, n = 1; cottonseed hulls, n = 1; soyhulls, n = 1; distillers dried grains with solubles (**DDGS**), n = 1; and TMR, n = 6] and 34 fecal samples were collected. All samples originated from dairy nutrition studies conducted at Ohio Agricultural Research and Development Center of The Ohio State University (Wooster, OH). Samples provided were from experiments in which all animal-based protocols were approved by The Ohio State University Institutional Animal Care and Use Committee (IACUC). Because procedures used in the current study did not require new research activities utilizing animals, a single new IACUC protocol was not required.

To isolate NDF residues, feed and fecal samples were dried at 60°C for 48 h and ground through a 1-mm sieve (Wiley Mill; Arthur A. Thomas Co.). Once ground, NDF residues were isolated using the Ankom technique (Ankom200 Fiber Analyzer, Ankom Technology Corp.). This assay was conducted in quadruplicate and included 0.5 g of sodium sulfite and 1 mL of α-amylase (Sigma A3306; Sigma-Aldrich). Neutral detergent fiber residues were then further ground manually using a mortar and pestle and once again dried at 60°C for 24 h. Samples were ground manually because of the limited amount of sample available and because mechanical grinding would lead to more sample loss. A bomb calorimeter (Parr 6400 Calorimeter, Parr Instrument Company) was used to determine concentration of GE. The bomb calorimeter was calibrated after 2 benzoic acid standards were within the range of 6,318 ± 18 Mcal/kg. Then, 0.2 g of the ground NDF residue was placed in a tared metal cap, followed by 0.4 g of mineral oil. Samples were set to rest overnight so that the mineral oil could completely soak the sample before being placed in the bomb calorimeter. All individual samples were analyzed in duplicate. Although sample amount was lacking for all samples, a subset of feed (n = 10) and fecal (n = 12) residues were also analyzed for ash content (943.05; AOAC International, 2000).

Differences in GE energy content between feed and fecal samples were tested using the TTEST procedure of SAS (version 9.4; SAS Institute Inc.). Using the UNIVARIATE procedure method within SAS data were screened for outliers, which were defined as those observations ± 2.5 standard deviations from the treatment means. One fecal NDF sample was removed because its GE concentration was more than 2.5 standard deviations below the mean.

The aim of this study was to evaluate analytical estimates of GE contained in NDF. Practically, this is of interest to the field of dairy nutrition because in estimating DE, the current NASEM (2021) employs a summative equation that uses an assumed GE concentration of nutritive entities that supply energy. In the case of NDF, lignin can be used to estimate the digestibility, but we speculated that because it inherently has a greater concentration of GE, the appearance of lignin in NDF could also influence analytical estimates of GE energy in this fraction. The GE concentration of NDF used in the NASEM (2021) is assumed to be 4.2 Mcal/kg, whereas in the current study, we observed this to be 4.03 ± 0.245 Mcal/kg. The lower estimate of the GE concentration was at least in part due to contamination of ash in the NDF residue (Higgs et al., 2015), which contributed mass but not GE (Weiss and Tebbe, 2019). In the current study, the average ash content of feed NDF residues was 1.83% on an NDF basis and when corrected for ash, GE from NDF is increased by 0.08 Mcal/kg (4.03 to 4.11 Mcal/kg). Van Soest et al. (1991) suggested either correcting NDF values for ash contamination or reporting dietary ash content when studying forages or other feeds because ash from soil contamination during harvesting methods may vary. In the current study sample mass was limited and all samples could not be analyzed for lignin, neutral detergent insoluble crude protein (**NDICP**), or ash. This information could have proven useful in identifying other nutrients responsible for the difference observed in GE concentrations. We also hypothesize that because lignin is indigestible and has a greater GE concentration than carbohydrate, that GE concentration would be greater for NDF in fecal residue than for NDF in feed residue. If this hypothesis should hold, we believed this could be a contributing factor to the variation in DE that has been reported (Tebbe et al., 2017). Surprisingly, the GE concentration between feed (4.03 ± 0.245 Mcal/kg) and fecal samples (3.94 ± 0.245 Mcal/kg) was not observed to be different (*P* = 0.23). Fecal NDF ash was 0.72 percentage units greater compared with feed NDF residues (1.83 and 2.55% NDF for feed and fecal NDF, respectively). Theoretically, this difference in ash content would account for a decrease of 0.03 Mcal/kg or 33% of the numerical difference between the observed difference in feed and fecal NDF GE. Similarly, the proportion of CP within the NDF fraction increase in fecal NDF residues from 3.10% NDF to 5.44% NDF. This 2.34% increase in CP in fecal NDF residues should account for an increase in GE concentration by 0.13 Mcal/kg assuming NDICP has the same GE concentration as feed protein (5.65 Mcal/kg, NASEM, 2021).

While not measured in the current study, the proportion of lignin within the NDF residue in fecal samples is expected to be greater than that in feed sample. For example, the typical corn silage NDF residue is approximately 10% lignin (NASEM, 2021), and according to Hindrichsen et al. (2006), the paired fecal NDF residue of corn silage should contain approximately 20% lignin. Using a GE concentration of 6.0 Mcal/kg, lignin in fecal NDF would contribute 1.20 Mcal per kg of total NDF, whereas lignin in feed NDF would contribute 0.60 Mcal per kg of total NDF. This difference was not observed in the current study when comparing GE concentrations of feed and fecal NDF residues, suggesting that NDICP and ash accumulation in fecal NDF do not account for the entirety of the expected difference in GE concentration for feed and fecal NDF residues, and we speculate that the nature of hemicellulose digestion might also be an important factor in explaining the lack of a difference in feed and fecal observed.

We interpret the lack of a difference in GE between feed and fecal as evidence that NDF is of similar GE concentration before and after total-tract digestion in the cow. Consequently, this likely means that the digestible proportions of NDF are similar in GE and current nutritional models appropriately use feed NDF GE concentrations to predict DE. However, the use of the current GE coefficient for feed NDF in models like the NASEM (2021) and CNCPS (6.55; https://cals.cornell.edu/animal-science/outreach-extension/publications-resources-software/cncps) may not be reflective of the true energy content. Specifically, the current study analytically determined the GE concentration of feed NDF to be 4.03 Mcal/kg, whereas nutritional models use coefficients of 4.20 Mcal/kg in their predictions of DE, resulting in an overprediction of DE estimates from NDF of about 4%. The use of a 4.03 Mcal/kg GE coefficient in nutrition models could be validated using a typical NDF profile and common GE concentrations for the primary constituents of the NDF profile. According to the NASEM (2021) feed library, the NDF in typical corn silage contain approximately 55% cellulose, 35% hemicellulose (as estimated as NDF minus ADF), and 10% lignin. Multiplying the relative proportion of these constituents by their respective GE concentrations would yield an overall feed NDF GE concentration of 4.02 Mcal/kg. Thus, calculating GE from measured feed composition could be a simple and accurate way to generate a model input of GE.

Hemicellulose could also be a contributing factor to variation in GE concentration feed NDF residues. The GE coefficients for xylan in the literature range from 3.04 (Dorez et al., 2014) to 3.25 Mcal/kg (Gorensek et al., 2019). This range in GE values is similar to the difference in the observed NDF GE concentration value from the current study (4.03 Mcal/kg) and the GE concentration used by the NASEM (4.20 Mcal/kg). Xylose can account for 30% to 90% of the sugars present in hemicellulose depending on analytical methodology and type of hemicellulose, with the remaining sugars consisting of glucose, galactose, arabinose, and fructose (Peng et al., 2019). The chemical composition of hemicellulose is also subject to variation depending on plant species and maturity (Wedig et al., 1987), further contributing to the variance around the true mean GE concentration of hemicellulose. Compared with the hexose sugars found in hemicellulose, pentose sugars are more reduced and therefore are of a lower GE concentration (Blaxter, 1989). There is a potential that fecal NDF could have been enriched in xylose and this could have been a contributing factor to the lower-than-anticipated observed fecal NDF GE concentration. If the true GE concentration of hemicellulose is approximately 3.04 to 3.25 Mcal/kg, the energetic contribution from feeds with NDF profiles rich in hemicellulose, like DDGS, will be overestimated by nutritional models. The extent of this overestimation would be affected by the proportion of these feeds being included in the diet. Additionally, the hemicellulose GE concentration values of Dorez et al. (2014) and Gorensek et al. (2019) were derived using hemicellulose from softwood trees because the extraction of hemicellulose from forages lacks a sound analytical procedure. The application of tree hemicellulose in feed energetics might be inaccurate. The heterogeneity and lack of laboratory methods to accurately precipitate feed hemicellulose hinder our understanding of how hemicellulose contributes to the energy concentration of NDF.

The current study observed a large degree of variation in the GE concentrations of NDF residues from different feeds (Table 1). For example, NDF residues isolated from soybean hulls averaged 3.95 Mcal/kg, whereas residues isolated from DDGS averaged 4.64 Mcal/kg. According to the feed NASEM (2021) feed library the average soybean hulls NDF residue contained 68% cellulose, 28% hemicellulose (as calculated by NDF minus ADF), and only 4% lignin. The low GE concentration of the NDF residue from soybean hulls is likely a function of a low concentration lignin in this feedstuff. However, using the feed library in the NASEM (2021), the average NDF residue isolated from DDGS contained approximately 32% cellulose, 52% hemicellulose (as calculated by NDF minus ADF), and 16% lignin. The high concentration of GE in NDF from DDGS is not likely to be a result of greater hemicellulose but could be a result of the high concentration of NDICP known to be in this feedstuff. The NDF residue from wheat straw and alfalfa hay also contained relatively high concentrations of GE concentrations, averaging 4.15 and 4.12 Mcal/kg, respectively. We speculate that this is a result of greater proportions of lignin within their NDF residues, which according to NASEM (2021) are 16% and 11%. Overall, the great degree of variation among GE concentrations of common feedstuffs in dairy rations suggests that a universal GE coefficient used by ration formulation software for total dietary NDF might be an oversimplification.

**Table 1:** Mean gross energy (GE) concentration (Mcal/kg) for feed and fecal NDF residues^1^ with SD shown in parentheses

Item	Sample type^2^		*P*-value
	Feed	Fecal	
Alfalfa hay	4.15 (0.078)	—	—
Corn silage	4.01 (0.053)	—	—
Cottonseed hulls	4.00	—	—
DDGS	4.64	—	—
Grass hay	4.19 (0.062)	—	—
Soybean hulls	3.95	—	—
TMR	3.85 (0.212)	3.94 (0.245)	—
Wheat straw	4.12	—	—
Average	4.03 (0.245)	3.94 (0.245)	0.23

1 Residues collected using the Ankom technique (Ankom200 Fiber Analyzer, Ankom Technology Corp.).

2 Alfalfa hay, n = 4; corn silage, n = 2; cottonseed hulls, n = 1; distillers dried grains with solubles (DDGS), n = 1; grass hay, n = 2; soybean hulls, n = 1; TMR, n = 6; wheat straw, n = 1; feed average (feed and TMR), n = 16; and fecal average, n = 34.

Although narrow in scope, this study provides information on important assumptions used to estimate energy by the NASEM (2021) model. Because the variation (coefficient of variation = 6.21%) in the GE concentration of NDF was observed to be high and this variation, whether real or due to analytical variation, negatively affected the statistical power of our tests and could contribute to a type II statistical error. Future research should seek to identify major sources of this variation, including the potential of interaction by feedstuff type. Given the limitations of the current study, future studies should (1) also measure the concentration of GE contained in NDF of more feeds, (2) have greater replication within a feedstuff (i.e., not analytical replication, but more feedstuffs and more representative samples within a feedstuff population) to determine if interactions by feedstuff exists, and (3) seek to measure the concentrations of contaminates such as CP and ash in NDF residue and measure their impact on GE. That the GE concentration was observed to be different than starch, a more uniform carbohydrate, is not surprising because in addition to lignin, cellulose, and hemicellulose, NDF residues could also contain some interfering protein and ash. In conclusion, being less than what is assumed, the energy supplied by digestible NDF may be lower than what is used by the NASEM (2021).
